# An integrated assessment of nitrogen source, transformation and fate within an intensive dairy system to inform management change

**DOI:** 10.1371/journal.pone.0219479

**Published:** 2019-07-23

**Authors:** Elisa Clagnan, Steven F. Thornton, Stephen A. Rolfe, Naomi S. Wells, Kay Knoeller, John Murphy, Patrick Tuohy, Karen Daly, Mark G. Healy, Golnaz Ezzati, Julia von Chamier, Owen Fenton

**Affiliations:** 1 Environmental Research Centre, Teagasc, Johnstown Castle, Co. Wexford, Ireland; 2 Groundwater Protection and Restoration Group, University of Sheffield, Sheffield, United Kingdom; 3 Free University of Bolzano, Faculty of Science and Technology, Bolzano, Italy; 4 Department of Animal and Plant Science, University of Sheffield, Sheffield, United Kingdom; 5 Centre for Coastal Biogeochemistry, School of Environment, Science and Engineering, Southern Cross University, Lismore, Australia; 6 Department of Catchment Hydrology, Helmholtz Centre for Environmental Research - UFZ, Halle (Saale), Germany; 7 Animal and Grassland Research and Innovation Centre, Teagasc, Moorepark, Co. Cork, Ireland; 8 Civil Engineering, National University of Ireland, Galway, Co. Galway, Ireland; Chinese Academy of Sciences, CHINA

## Abstract

From an environmental perspective optimised dairy systems, which follow current regulations, still have low nitrogen (N) use efficiency, high N surplus (kg N ha^-1^) and enable *ad-hoc* delivery of direct and indirect reactive N losses to water and the atmosphere. The objective of the present study was to divide an intensive dairy farm into N attenuation capacity areas based on this *ad-hoc* delivery. Historical and current spatial and temporal multi-level datasets (stable isotope and dissolved gas) were combined and interpreted. Results showed that the farm had four distinct attenuation areas: *high N attenuation*: characterised by ammonium-N (NH_4_^+^-N) below 0.23 mg NH_4_^+^-N l^-1^ and nitrate (NO_3_^-^-N) below 5.65 mg NO_3_^-^-N l^-1^ in surface, drainage and groundwater, located on imperfectly to moderately-well drained soils with high denitrification potential and low nitrous oxide (N_2_O) emissions (av. 0.0032 mg N_2_O-N l^-1^); *moderate N attenuation*: characterised by low NO_3_^-^-N concentration in drainage water but high N_2_O production (0.0317 mg N_2_O-N l^-1^) and denitrification potential lower than group 1 (av. δ^15^N-NO_3_^-^: 16.4‰, av. δ^18^O-NO_3_^-^: 9.2‰), on well to moderately drained soils; *low N attenuation—area 1*: characterised by high NO_3_^-^-N (av. 6.90 mg NO_3_^-^-N l^-1^) in drainage water from well to moderately-well drained soils, with low denitrification potential (av. δ^15^N-NO_3_^-^: 9.5‰, av. δ^18^O-NO_3_^-^: 5.9‰) and high N_2_O emissions (0.0319 mg N_2_O l^-1^); and *low N attenuation—area 2*: characterised by high NH_4_^+^-N (av. 3.93 mg NH_4_^+^-N l^-1^ and high N_2_O emissions (av. 0.0521 mg N_2_O l^-1^) from well to imperfectly drained soil. N loads on site should be moved away from low attenuation areas and emissions to air and water should be assessed.

## Introduction

Reactive nitrogen (N) surplus in agricultural watersheds accumulates in the rooting zone of soils due to low nutrient use efficiencies [1]. This N storage can affect connected water quality for long periods where the biogeochemical time lag is long [2]. Therefore, minimising the N load in areas with high biogeochemical time lags and connectivity to water bodies is an important measure for N attenuation. Nitrogen loading originates from a mixture of animal waste and fertiliser inputs, comprising both inorganic (urea, calcium ammonium nitrate (CAN)) and/or organic (dairy soiled water (DSW), manure, slurry and urine) components. As the subsurface is heterogeneous surplus N can be transformed at different rates through mainly biological processes within the N cycle and especially nitrification and denitrification, in which ammonium (NH_4_^+^-N) is oxidised to nitrate (NO_3_^-^-N) and then reduced to di-nitrogen gas (N_2_) [3]. This and other pathways (e.g. nitrification, DNRA (dissimilatory nitrate reduction to ammonium), anammox) are composed of sequential reactions, with the production and possible release of intermediate and undesirable N compounds to the environment, e.g. NO_3_^-^-N, nitrite (NO_2_^-^-N) and nitrous oxide (N_2_O). Current policy instruments, under the EU Water Framework Directive [4] treat the farm as a homogeneous soil block where there is no acknowledgement of the *ad-hoc* delivery of direct and indirect reactive N losses to water and the atmosphere. However, the ability of the soil/subsoil and underlying geology to attenuate this N surplus is heterogeneous. Dividing an intensive dairy farm into N attenuation capacity areas based on actual *ad-hoc* delivery could minimise future losses of N.

Clagnan et al. [5,6] studied N attenuation from plot to field scales using biogeochemical, gas and isotope techniques, ranking poorly drained sites (artificially drained and un-drained) with respect to their natural attenuation capacity. Results showed that deep groundwater drainage systems did not negatively affect the N attenuation capacity of a soil. A concept of “net” water origin, N source, transformation and fate was developed using water samples from borehole screen intervals and end-of-pipe locations [5,6]. This concept enables interpretation of water origin and identifies a clear migration pathway for that signature (e.g. precipitation, groundwater and a mixed enriched signal inferring migration along a pipe or ditch) over much larger areas, and thus highlights connectivity or dis-connectivity of N sources, transformations, pathways and receptors. Until now this concept has never been examined at farm-scale (125 ha).

Research on soil functions has indicated that the N water purification function of soil, identified by Schulte et al. [7] as “the amount of denitrification required to ensure that the N surplus leaving the rooting zone does not lead to excess groundwater N concentrations”, is higher in poorly drained (low permeability) and lowest in freely drained (high permeability) soils [8,9]. The N water purification function of different agricultural soils has a significant impact on reducing/controlling N concentration and the ratio among N species, with denitrification being the main process of N removal [10,11]. N speciation and attenuation capacity is regulated by many other environmental factors, e.g. substrate concentrations, plant coverage, management and weather [12].

The role of artificial drainage on N transfer, transformation and migration on intensive dairy farms with variable soil types and drainage classes remains unclear. A drainage pipe installed within the sub-soil is likely to connect areas of “low” and “high” denitrification potential, depending on soil functional characteristics [7]. The end-of-pipe water sampled reflects the composite attenuation capacity of the subsoil draining into this pipe. However, these systems may reduce N transformation potential by creating unsuitable conditions for *in situ* denitrification (e.g. higher oxygen concentration, lower water saturation and shorter residence time), leading to greater NO_3_^-^-N losses. Equally, zones of high soil N attenuation capacity may be by-passed by the drainage system. Other N species, such as NH_4_^+^-N, NO_2_^-^-N and N_2_O, are seldom considered and there is a need to examine if drainage system pathways in heterogeneous soils can utilise areas which support N attenuation capacity, or by-pass and nullify this capacity, with increased reactive N losses from the system. There have been limited investigations of NO_3_^-^-N distributions in shallow groundwater systems under such intensive dairy farms [8,13]. On the present study site, Baily et al. [14] used NO_3_^-^-N natural isotopic abundances for three sampling periods (April, August and Dec) to deduce the role of denitrification in soil N dynamics. Other studies [10,15] estimated such N-losses to be approximately 106 kg N ha^-1^, explained by hydrological and geochemical factors (e.g. availability of dissolved oxygen (DO) concentration and redox potential (Eh)). While these studies offered insight into the fate of NO_3_^-^-N in shallow groundwater, they did not increase knowledge pertaining to N provenance, multi-level spatial distribution or the transformation of N along shallow pathways and deep pathways or the ultimate fate of the lost N. Nutrient concentrations, hydrochemical parameters (e.g. pH, DO, Eh) and soil properties (e.g. saturated hydraulic conductivity, k_s_) can provide qualitative evidence of spatial and temporal variation in N-transformation processes and the soil conditions supporting these. Additional gas analyses may increase the understanding of local N-transformation processes, which can be quantified (in term of sinks and sources of N_2_O) by the release of their final gaseous products (i.e. N_2_ and N_2_O). However, since multiple N-transformation processes can contribute to N_2_ and N_2_O production, further analysis using isotopic abundances can help elucidate the different N sources and transformation processes (e.g. [16]).

The objective of the present study was to examine how dairy farm N surplus, N source and N attenuation on a heterogeneous landscape can be managed to reduce N loads to vulnerable areas, thereby protecting water quality into the future. To meet this objective, an extensive dataset was assembled over a 12-year period, from a combination of previous and new fieldwork.

## Materials and methods

### Study site

**Fig 1:**
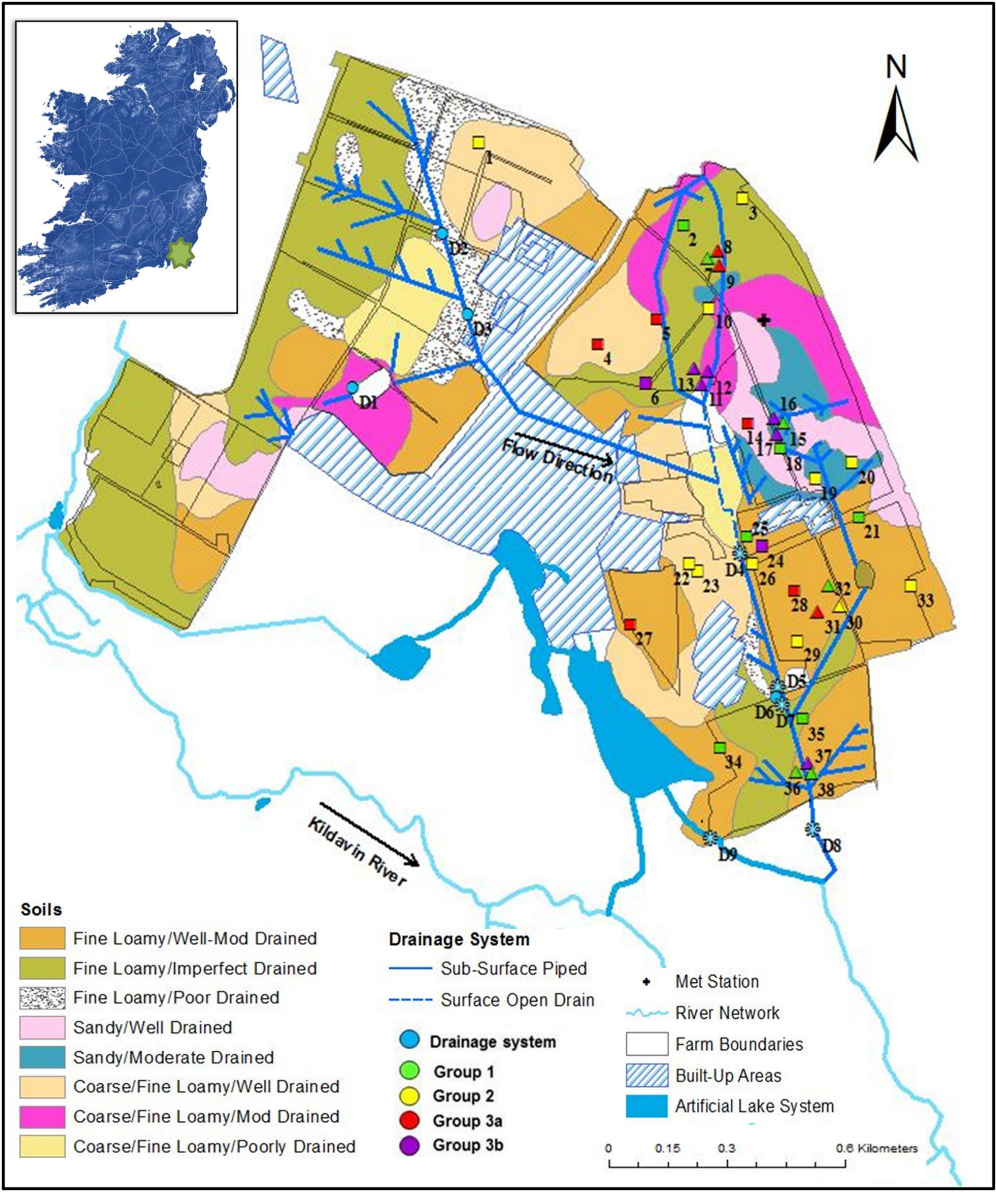
Map of the intensive farm merging soil texture, drainage class, position of the surface and subsurface drainage networks including the lake system and outlet and groundwater monitoring locations (Squares indicate piezometers, multilevel boreholes are indicated by triangles, end-of-pipe locations by circles and open ditch locations by asterisks).

The Teagasc Johnstown Castle, Co. Wexford, (52°17'30″N, 6°29'50″W) (Fig 1) study site comprises two units of grassland, one of 72.9 ha, termed “down-gradient”, and an “up-gradient” of 50.8 ha separated by a roadway. The up-gradient subsurface drainage system is connected to the down-gradient system through an underground connector pipe. The farm, considering the two grassland units together, is intensive and operates at 3.1 Livestock units per hectare (LU ha^-1^). Nitrogen inputs arise from urea (spread February to April), calcium ammonium nitrate (CAN) (May to September) and manure (spring) for a total of 257 kg N ha^-1^ inputs of inorganic fertiliser and 103 kg N ha^-1^ of organic (5 years average). The central area of the dairy farm receives dairy soiled water (DSW, consisting of rainwater, yard and milk parlour washings) from Feb to Oct through a rotational irrigation system (Fig 1, former spreading area: plot with locations 11 and 14; current spreading area: between the Met station and location 19). To quantify the N surplus that could affect the drainage system, farm partial N balances for each year were calculated as per Treacy et al. [17], utilising stocking rates, N inputs (inorganic and organic fertilisers and concentrate feed (volume and composition)) and N exports (milk production (volume and composition) and slurry). Cows were milked twice daily (07·30 h and 15·30 h), with milk yield (kg) registered for each cow each time. The milk composition (fat, protein and lactose concentrations) for each cow was tested on a successive morning and evening every two weeks using a Milkoscan 203 (Foss Electric DK-3400, Hillerød, Denmark). Milk solids were calculated using the method of Tyrell and Reid [18]. For both milk and concentrates fed the N value is averaged across the farm. This region has an average annual air temperature of 10°C (1981–2011), average annual rainfall (1981–2011) is 1037.5 mm with maximum intensity between Sep and Nov. There is an *in situ* synoptic meteorological station (Fig 1) on site which records daily rainfall, wind speed and hours of sunshine. This data was inputted into the grassland hybrid soil moisture deficit (HSMD) model of Schulte et al. [19] to estimate effective drainage (ED, mm day^-1^). To examine differences in ED modelling was conducted for well-, moderately- and poorly-drained soils using data from 2008 to 2017. Soil texture varies from fine loam to clay loam (Brown Earth, Gleyic Cambisol with Irish Sea till origin [20], Fig 1), with small areas of sandy textured soils. Subsoil is of moderate permeability (0.2–10 m; 5 x 10^−8^ m s^-1^ <k_s_< 5 x 10^−4^ m s^-1^) but can be interspersed with high permeability gravel and/or sand lenses [8, 21]. Unsaturated zone hydrologic time lag is from 1 (shallowest areas) to 3 (deepest areas) years with probability analysis indicating 1.5 years 85% of the time [14], presenting a mismatch between best management practice at farm level, when nutrients are lost, stored and mineralised and when leaching affects N concentrations along subsurface pathways. The average water table depth on site from 2005–2014 is 2.8 (±1.7) m below ground level (bgl) (deepest from July to Sep i.e. 3.0 m bgl and shallowest from December to February i.e. 2.3 m bgl). The low permeability bedrock is Pre-Cambrian greywacke mixed with quartzite at a depth of 10–12 m (k_s_, 3.6 x 10^−6^ m s^-1^), containing a poorly productive aquifer, which is classified as receiving 1 to 50 mm yr^-1^ [14].

### Surface and subsurface monitoring network

A large surface and subsurface monitoring network was collated in GIS and a map developed for the purpose of the study. The open ditch network and groundwater monitoring components of the system were well documented, but the subsurface drainage system remained unmapped. Examination of historical maps and discussions with farm staff identified likely positions of in-field drains, which were confirmed by field work (Fig 1, S1 Table). Both up- and down-gradient units contain poorly-imperfectly drained soils (79% and 28%, respectively) (Fig 1). These areas are all artificially drained by an in-field pipe network of 10.1 km (Fig 1) composed of either corrugated slotted plastic (80 or 100 mm at 0.5–1 m depth) or concrete non-perforated pipes (600 mm at 1 m depth act as connectors).

The components of the surface and subsurface drainage system are as follows: 1) *Up-gradient component*: a herring bone drainage network (80 mm, variable depth) conveys drainage water from an area of 24 ha, to an underground outlet (D2, Fig 1) and joins with another outlet at D3, which passes to a junction also fed by a drainage system passing through D1. This composite migrates in a fully cased concrete pipe and eventually discharges into the open ditch in the down-gradient unit. 2) *Down-gradient component including open ditch*: the subsurface drain that transfers water from locations 2, 3, 7, 8, 9 and 10 is an *ad-hoc* drain developed over time with many unknown extensions and discharges to the start of the open ditch. A short herring bone system discharges water into the open ditch adjacent to location 14. The open ditch extends from 11 to D4, before being piped underground to the outlet at D8 (Fig 1). There is an access point in the underground section at D5-6-7. Individual drains connect to the underground part of this primary drain at location 30 (fully cased draining a marl pond) and 36. 3) *Unconnected components*: an in-field herring bone drain in the area of 15, 16 and 17 flows in the opposite direction to the open drain and has an offsite outlet, as does the system around 19 and 20. Any discharge from the nearby artificial lake system (D9) does not affect D8. Prior to the 2014 sampling campaign, water sampling locations were added to this system, i.e. three positions along the subsurface drainage system (D1, D2, D6), one position along the surface stream (D5) and three within the groundwater network (1, 22, 23) (Fig 1).

### Historical data

Twelve years of data for the down gradient dairy farm were collated from the groundwater monitoring network (S1 Table, No 5). The groundwater monitoring system consists of three components: 1) five sets of multilevel boreholes (Fig 1) representing three depths: subsoil (4.0–7.5 m bgl), bedrock (16.8–23.0 m bgl) and the subsoil-bedrock interface (11.0–13.0 m bgl); 2) a network of piezometers and boreholes (Fig 1), with screen depths from 1.95 to 8.95 m bgl installed to sample shallow groundwater within the subsoil layer; 3) a single borehole drilled to 37 m bgl was installed to sample deep groundwater (18, Fig 1).

Fieldwork was conducted from Dec 2005 to June 2017 (S1 Table, No 5). During this period, grab samples were taken from open ditch and end-of-pipe locations indicated in Fig 1. Groundwater samples were collected after purging three volumes [22] with a peristaltic pump (Model 410, Solinst Canada Ltd.) and teflon outlet, connected to a flow cell fitted with an In-Situ Multi-parameter Probe (pH, temperature (T), electrical conductivity (EC), DO and Eh) (In Situ Inc., USA). Duplicate 50 ml samples were collected in HDPE screw top bottles and filtered with 0.45 μm cellulose acetate filters (Sartorius Stedim Biotech GmbH, Germany). The water table depth was measured with an electronic dipper (Van Walt Ltd., Surrey, UK).

### Current data

Fieldwork was undertaken in Sep 2014 and June 2017 to collect water samples for all locations in Fig 1. These two sampling campaigns were carried out to integrate different techniques (i.e. stable isotope and dissolved gas analyses (only for Sep 2014) and all elements of the water continuum (i.e. shallow and groundwater, in field drainage and open ditches). These campaigns also deduced the stability of the N signatures and dissolved gases on site within shallow water which was highlighted by Baily et al. [14] and Jahangir et al. [10,11] across the multilevel borehole network (S1 Table). Water was sampled using a bladder pump (flow rate of 100 ml min^-1^) with a Teflon outlet tube (diameter: 0.6 cm) (Geotech Environmental Equipment, Inc., USA) following a low-flow micro-purging protocol [22]. This pump minimises sample mixing and degassing [10]. Otherwise a peristaltic pump and 20 ml syringe connected to a Teflon tube (diameter: 0.5 cm) with 3-way stopcock was used. Triplicate 50 ml surface water samples from pipes or open drains were collected manually in HDPE screw top bottles and stored at 4°C until analysed. One replicate was filtered in the field (0.45 μm cellulose acetate filter). As per historical data an electronic dipper and In-Situ Multi-parameter Probe were used in the field.

### Water and gas analyses

Water samples (both current and historical) were analysed within two weeks of collection for the following: NO_2_^-^-N, NH_4_^+^-N, Total Oxidised Nitrogen (TON) and dissolved organic carbon (DOC) were quantified using an Aquakem 600 Discrete Analyser (Aquakem 600A, 01621 Vantaa, Finland). Concentrations of NO_3_^-^-N were calculated by subtraction of NO_2_^-^-N from TON (NO_3_^-^-N + NO_2_^-^-N). Total Nitrogen (TN) was determined by alkaline persulfate oxidation [23].

For current fieldwork, duplicate water samples were collected (all locations) for excess-N_2_ estimation in 12 ml exetainers (LabcoWycomb Ltd., UK) after overflow of 10 ml and duplicate groundwater samples were collected for the quantification of dissolved N_2_O, carbon dioxide (CO_2_) and methane (CH_4_). The analyses and calculation of gaseous concentrations for excess-N_2_, dissolved N_2_O, CO_2_ and CH_4_ were carried out as per Clagnan et al. [5,6]. The indirect N_2_O-N emission factor (N_2_O-N EF_5_g) for groundwater was calculated from dissolved-N_2_O and N-inputs, as per Weymann et al. [24] using the following equation: EF_5_g(1) = (N_2_O-N)/(N_2_O-N + N_2_-N + NH_4_^+^-N + NO_3_^-^-N + NO_2_^-^-N + DON).

### Stable isotope analysis

In Sep 2014 and June 2017 groundwater samples (40 ml) were collected on two dates, filtered in the field through 0.2 μm polyethersulfone filters (Sartorius Stedim Biotech GmbH, Germany) and stored at -20°C in 50 ml polyethylene screw cap tubes. Samples were analysed (Dept. of Catchment Hydrology, UFZ, Germany) for the isotopic composition of NO_3_^-^ (^15/14^N and ^18/16^O), NH_4_^+^ (^15/14^N), and H_2_O (^2/1^H and ^18/16^O). Gas exetainers of 12 ml were additionally used for dissolved-N_2_O (^15/14^N and ^18/16^O). Isotope values are reported in δ‰ relative to international standards (AIR for N and VSMOW (Vienna Standard Mean Ocean Water) for O and H). Water δ^18^O and δD (δ^2^H) signatures for H_2_O were analysed in accordance with [5,6]. The results are interpreted according to a modified Rayleigh equation: *f* = 1—*e*^(δs–δs0)/ε^, where *f* is the fraction of substrate remaining, ε is the isotope enrichment factor, and δs and δs0 are the isotopic composition of the residual substrate and initial substrate, respectively. The δ^15^N-NH_4_^+^ was measured in subsurface locations with detectable NH_4_^+^-N concentration. The δ^15^N signature for NH_4_^+^ was obtained as in Zhang et al. [25]. The δ^15^N-NO_3_^-^ and δ^18^O-NO_3_^-^ were obtained as in [26]. The δ^15^N and δ^18^O composition of the produced N_2_O (plus that of the dissolved N_2_O samples) were measured as in [5].

### Statistical analysis

Different methods (t-test, oneway ANOVA and Tukey's HSD test (IBM SPSS Statistics version 24)) were used to determine possible correlations and differences between nutrient, isotopic, and gaseous data and other hydrochemical parameters within the management groups.

## Results and discussion

### Partial N balance

Annual total N-inputs were estimated 364 kg N ha^-1^ (296 kg N ha^-1^—fertilizers, 68 kg N ha^-1^—feed concentrates) whereas national average for intensive farms is 223 kg N ha^-1^ ([17]) (Table 1). Irrigation N was composed of slurry and DSW and it was accounted as organic fertilisers. In this area, between 2013–2014, the annual sum of wet and dry deposition was 6 and 8 kg ha^-1^ per year (2–4 kg ha^-1^ per year of NH_4_^+^ for wet and dry deposition, respectively) [27]. Mineralization is a larger N source than ammonia deposition but variable and dependant on soil and climatic conditions. Murphy et al. [28] found that on sandy loam soils, with 6 kg ha^-1^ N deposition, net N mineralisation rates of 156 kg ha^-1^ per year occurred while of 161 kg ha^-1^ per year on clay loam.

**Table 1:** Five years annual N balance for the farm.

Year	2011	2012	2013	2014	2015	2016	2017	Average
Stocking Rate (per ha)	3.4	3	3.1	3	3.1	2.92	2.94	3
Grazing season (days)	238	215	244	256	263	236	242	242
**N Inputs (kg N ha^-1^)**								
Inorganic Fertilizer	232	270	266	262	256	248	253	255
Organic Fertilizer	39	44	36	38	41	44	43	41
Feed Concentrates	80	58	60	58	61	78	82	68
Total	351	372	362	358	358	370	378	364
**N Exported from the system (kg N ha^-1^)**								
Milk	132	124	122	123	123	118	123	124
Slurry	19	17	15	16	15	16.5	16	16
Total	151	141	137	139	138	135	139	140
**N Balance (kg N ha^-1^)**								
Surplus	200	231	225	219	220	236	239	224
N efficiency	43	38	38	39	39	36	37	38
**Milk production**								
Volume	23.3	22	21.5	21.7	21.9	20.6	21.5	22
Milk solids	1.78	1.63	1.61	1.65	1.67	1.54	1.59	2
**Total denitrification (N_2_O-N + excess N_2_-N (mg N l^-1^))** [15]								
Subsoil	1.76 ± 0.04							
Bedrock-interface	2.64 ± 0.43							
Bedrock	2.50 ± 0.33							
Site mean	2.30 ± 0.27							

With an N-output of 140 kg N ha^-1^ exported in milk and slurry, the estimated N-surplus is 224 kg N ha^-1^, which is stored in soil, used by the plant, lost from the system through leaching and runoff, or lost in the atmosphere as N_2_, N_2_O or as gaseous ammonia [29]. Ammonia emissions vary depending on climatic conditions during N application and applied N source; Misselbrook et al. [30] calculated that on English soils the emission factor from slurry and DSW is 34% of total ammonia N applied and 6% of total ammonia N applied, respectively.

### Spatial and temporal variation in aqueous N-species

**Fig 2:**
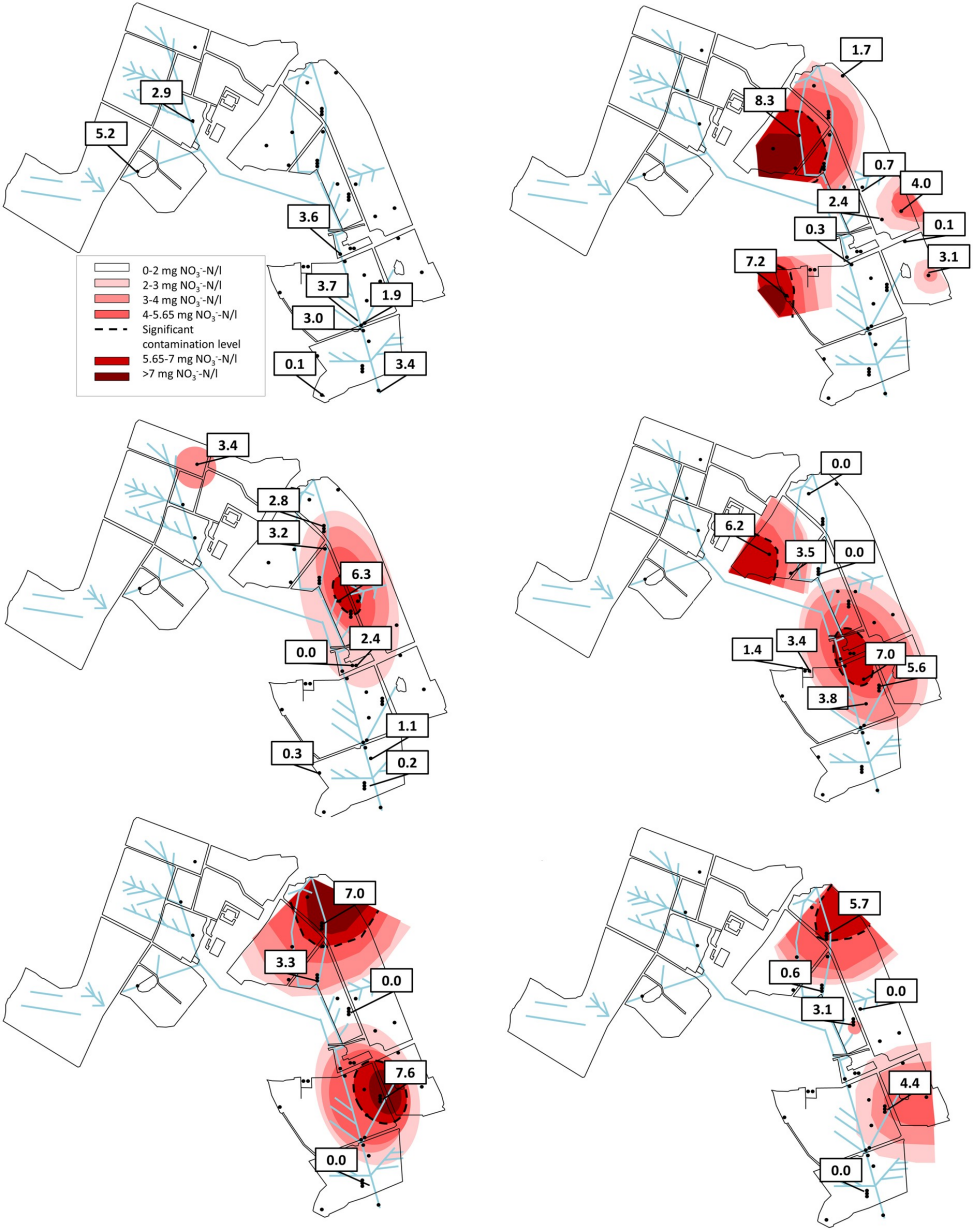
Depth specific N_3_^-^-N concentration as per the sampling campaign in Sep 2014. Top left: drainage system, top right: 2.95–4.5 m bgl, middle left 4.5–6 m bgl, middle right 6–9 m bgl, bottom left 11–13 m bgl, bottom right: below 16 m bgl.

Nitrate varied spatially from 25.3 mg NO_3_^-^-N l^-1^ to below detection. The overall average is low e.g. 4.5 mg NO_3_^-^-N l^-1^ in 2005, 3.0 mg NO_3_^-^-N l^-1^ in 2010, 3.6 mg NO_3_^-^-N l^-1^ in 2015 or 3.3 mg NO_3_^-^-N l^-1^ in 2017 (S1 Fig). In Sep 2014, location 1 (dominated by low permeability soil) had a NO_3_^-^-N concentration of 3.4 mg NO_3_^-^-N l^-1^, whereas in the down-gradient unit (variable soil permeability) three distinct shallow groundwater signatures emerged: a) in the north (locations 4–9), shallow samples ranged from 6.2 to 8.3 mg NO_3_^-^-N l^-1^, and deeper layers ranged from 5.7 to 7.0 mg NO_3_^-^-N l^-1^; b) well drained soils close to the sampling location 14 (see Fig 1, S2 Table) where concentrations ranged from 4.0 to 6.3 mg NO_3_^-^-N l^-1^; and c) central part of the farm (surrounding location 30) on a well-moderately drained soil exhibited concentrations up to 7.6 mg NO_3_^-^-N l^-1^ (Fig 2). The deepest well on the farm (location 18, depth: 37 m bgl) had a concentration of 3.07 mg NO_3_^-^-N l^-1^ and below NO_3_^-^-N maximum acceptable concentration (MAC), indicating the vertical extent of the NO_3_- plume to be around 16 m. There was no elevated concentration of NO_3_^-^-N in boreholes at the south end border of the farm (36–38) on imperfectly drained soil (Figs 1 and 2). At all times NO_3_^-^-N discharging from the drainage system was consistently low at <5.65 mg NO_3_^-^-N l^-1^ (the contamination threshold recommended by [31,32]).

**Fig 3:**
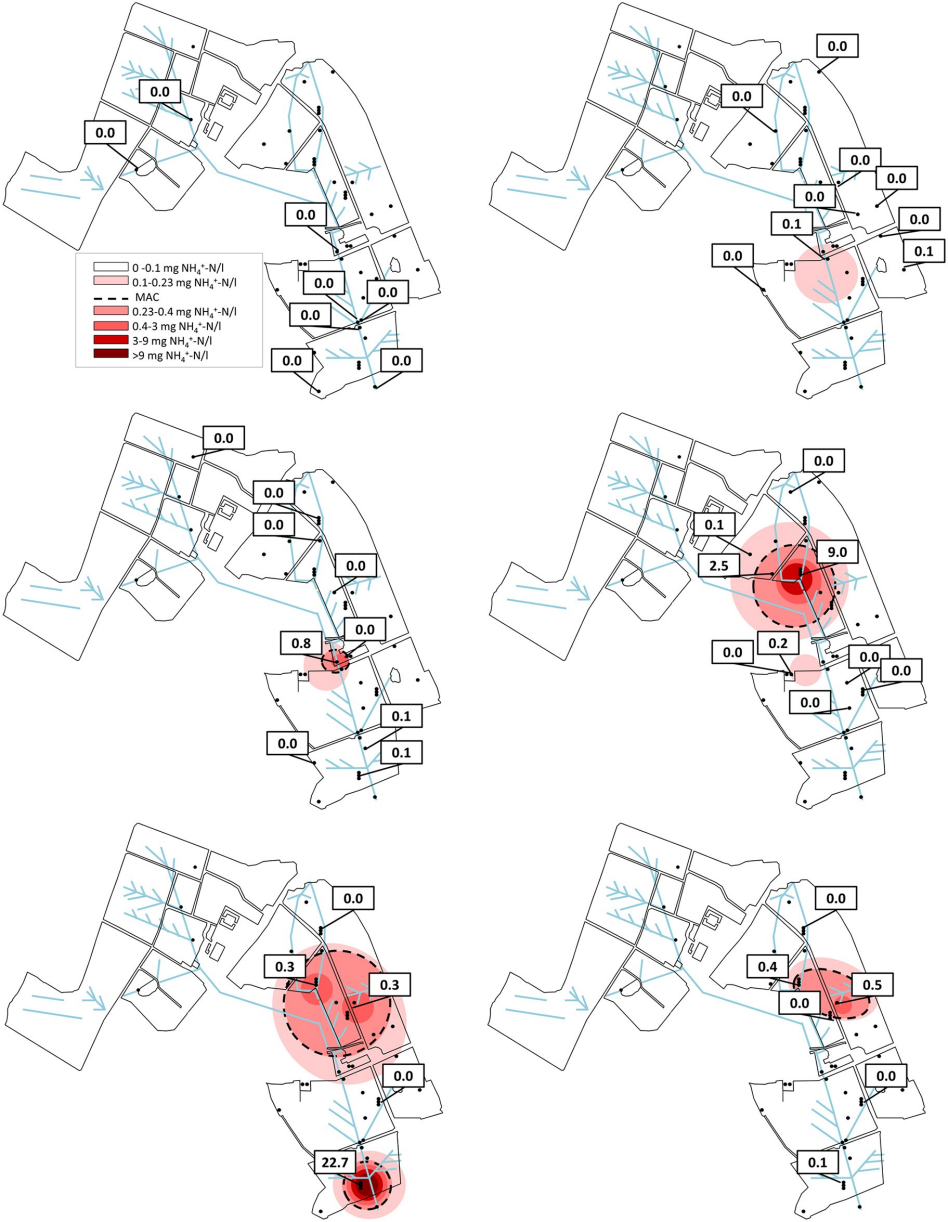
Depth specific NH_4_^+^-N concentration as per the sampling campaign in Sep 2014. Top left: drainage system, top right: 2.95–4.5 m bgl, middle left 4.5–6 m bgl, middle right 6–9 m bgl, bottom left 11–13 m bgl, bottom right: below 16 m bgl.

Ninety percent of locations had at least one sporadic concentration above the NH_4_^+^-N MAC over the years (2005–2017). End of pipe samples from the drainage system averaged at 0.01 mg NH_4_^+^-N l^-1^. In Sep 2014 the groundwater average was 0.98 mg NH_4_^+^-N l^-1^ (NH_4_^+^-N MAC: 0.23 mg NH_4_^+^-N l^-1^ [32]) mainly due to three elevated locations. The highest of these 22.7 mg l^-1^ occurred at location 37 (S1 Fig). Elevated locations typically occurred > 4 m bgl and at central locations (Fig 3).

### Dissolved-N_2_O and excess-N_2_

Dissolved gas (dissolved-N_2_O and excess-N_2_) results varied spatially and with depth. In water samples, the excess-N_2_ concentration averaged 2.28 mg N_2_-N l^-1^, with higher values at the interface and bedrock layer, while dissolved N_2_O was highest in the subsoil i.e. 0.024 mg N_2_O-N l^-1^ [15].

In Sep 2014, the average farm excess-N_2_ was 1.90 mg N_2_-N l^-1^ (max. 82 mg N_2_-N l^-1^; min. 0.0004 mg N_2_-N l^-1^) and the average drainage system was 0.076 mg N_2_-N l^-1^ (max, 0.18 mg N_2_-N l^-1^; min, 0.006 mg N_2_-N l^-1^ (S2 Fig, S2 Table). The up-gradient unit was characterised by emissions of 0.54 mg N_2_-N l^-1^. In the down-gradient area at shallow depth, excess-N_2_ was elevated in well- to moderately-drained soils, with peak concentrations of 3.36 mg N_2_-N l^-1^ at 2.95–4.5 m bgl and 4.32 mg N_2_-N l^-1^ at 4.5–6 m bgl. Up-gradient imperfectly drained soils had elevated concentrations (4.12 mg N_2_-N l^-1^) at 6–9 m bgl and these were higher in central parts of the farm (6.52 mg N_2_-N l^-1^ and 6.82 mg N_2_-N l^-1^) at 11–13 and 16 m bgl, respectively (S2 Fig). Dissolved-N_2_O averaged 0.03 mg N_2_O-N l^-1^ (max. 0.036; min. 0.0002 mg N_2_O-N l^-1^). The farm value for EF_5_g(1) was 0.0039 mg N_2_O-N/mg N input, compared with the IPCC default value of 0.0025 mg N_2_O-N/mg N input for groundwater N_2_O emissions. Both drainage and surface water emissions were below this default with only one in-field drain (D1, 0.0057 mg N_2_O-N/mg N input) had a higher value (S3 Fig). The up-gradient unit was characterised by a high emission factor (0.0243 mg N_2_O-N/mg N input), while the down-gradient unit (0–4.5 m bgl) had piezometers exceeding default values in the central area, reaching a maximum of 0.0081 mg N_2_O-N/mg N on well to moderately drained soil. At intermediate depths (4.5–9 m bgl) values were above the default values towards the north (6, 0.0325 mg N_2_O-N/mg N input) and south (23, 0.0114 mg N_2_O-N/mg N input; 37, 0.0097 mg N_2_O-N/mg N input). In contrast to excess-N_2_, N_2_O decreased with increasing depth, with almost no piezometer above the default values below 11 m bgl (S3 Fig).

A wide range of dissolved N_2_O vs. total emissions (N_2_O + N_2_) was found, which suggests a variable rate of denitrification. If the denitrification rate is high keeping the NO_3_^-^-N concentration below the contamination threshold, excess-N_2_ is released due to completion of the process. Conversely, high dissolved-N_2_O occurs where denitrification is limited [3]. Nitrification contributes to N_2_O production with stable isotopes (δ^18^O and δ^15^N) of N_2_O elucidating discrepancies in gas production and identifying N_2_O sources [33–36]. N_2_O-N was produced *in situ* and correlated with water table depth and k_s_ [13,15,21]. From Snider et al. [34] the expected range of δ^18^O-N_2_O produced by nitrification-denitrification on site was 0–20‰ (S4 Fig), which includes 75% of the piezometers. Piezometers with a relative enrichment of δ^18^O-N_2_O above these values are presumably only influenced by N_2_O reduction via denitrification. In addition, using the δ^18^O-N_2_O and δ^15^N-N_2_O ranges reported by Li et al. [37] and in particular the δ^15^N-N_2_O enrichment values, almost all locations have a N_2_O signature characterised by relative enrichment in δ^15^N-N_2_O, which reflects enrichment in the NH_4_^+^ source due to its consumption by microbial processes.

### Investigating connectivity

**Fig 4:**
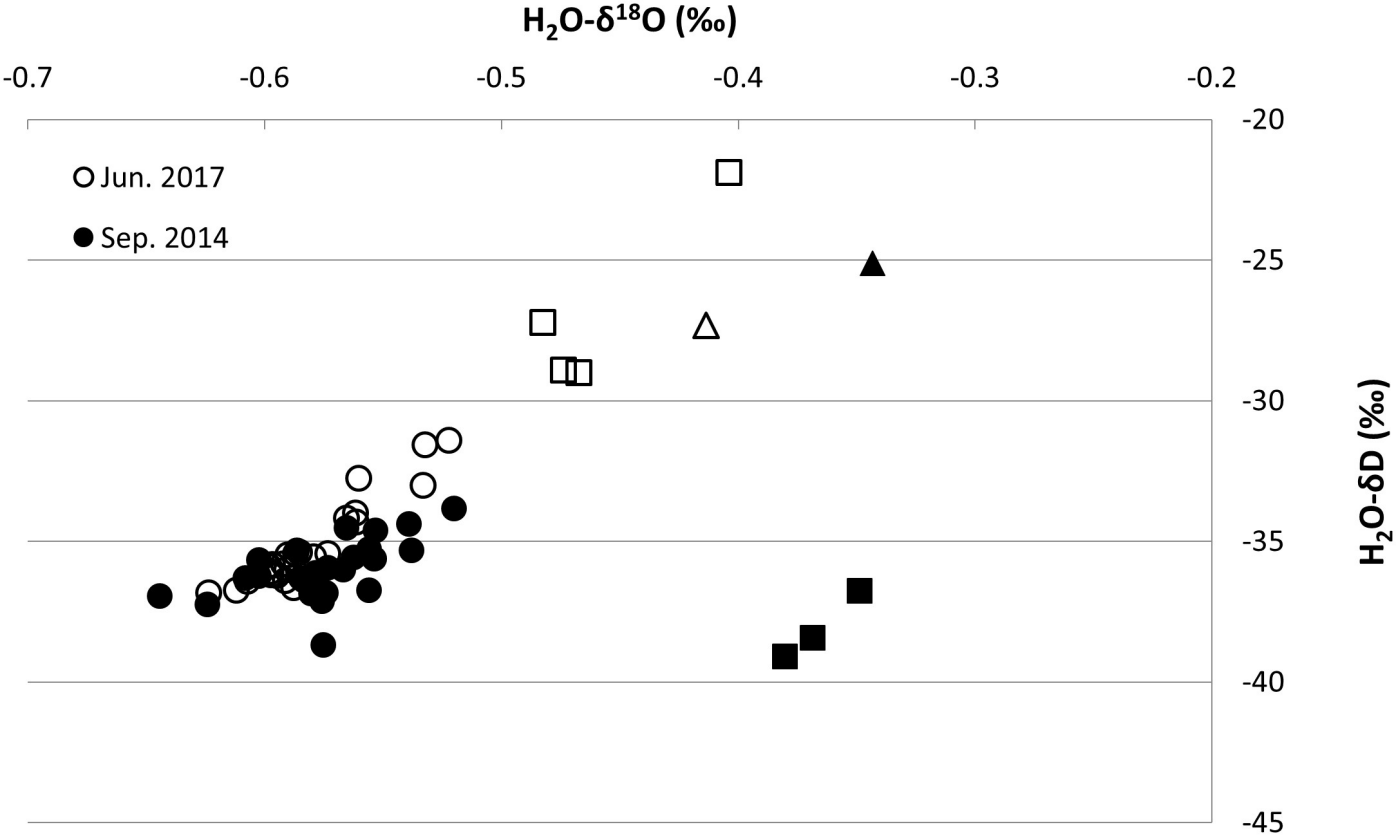
δ^18^O-H_2_O versus δD-H_2_O values for samples collected in Sep 2014 and June 2017 at the sampling locations. Circles indicate groundwater and infield drain samples, squares indicate open ditch samples while triangles indicate samples from the lake system.

Stable isotope compositions for all water samples showed low spatial variability, with values between -6.4 and -3.4‰ for H_2_O-δ^18^O and between -39.1 and -25.1‰ for H_2_O-δD in September 2014. Similarly, values ranged between -6.2 and -4.0‰ for H_2_O-δ^18^O and between -36.8 and -21.9‰ for H_2_O-δD in June 2017 (Fig 4). To examine the groundwater interaction with the drainage system, water stable isotope values were compared with the Global Meteoric Water Line (GMWL, δD = 8*δ^18^O+10) [38] to infer their composition and provenance (Fig 4). Farm values had a lower slope than the GMWL. Water samples (from screened intervals of piezometers and boreholes) had stable isotopic values close to the GMWL. Some sub-surface locations had the same water isotope values as many groundwater boreholes and piezometers. However, for other locations, e.g. along the surface open drain, there was an enrichment in δ^18^O but not δD relative to the GMWL in 2014, while in both δ^18^O and δD in 2017 (Fig 4), therefore showing a second signature due to evaporation in the open drain. A third signature from the lake system exhibited enrichment in both δ^18^O and δD-H_2_O (Fig 4).

Groundwater flow direction is from north to south, mirroring topography [14]. The isotopic composition of groundwater samples collected in Sep 2014 plots on a lower slope of the GMWL identified by Darling et al. [39], indicating a relative enrichment consistent with the high-humidity climate of the British Isles and findings of Gibson et al. [40]. Samples from the end-of-pipe and groundwater had the same signature, suggesting a common origin and interaction. However, other points in the open ditch were enriched in δ^18^O or in both δ^18^O and δD caused by migration and accentuated evaporation [41]. Overall, this indicates that water from a single source is connected along a groundwater, in-field pipe and open drain continuum.

### Spatial variation in nitrate δ^18^O and δ^15^N in water samples

**Fig 5:**
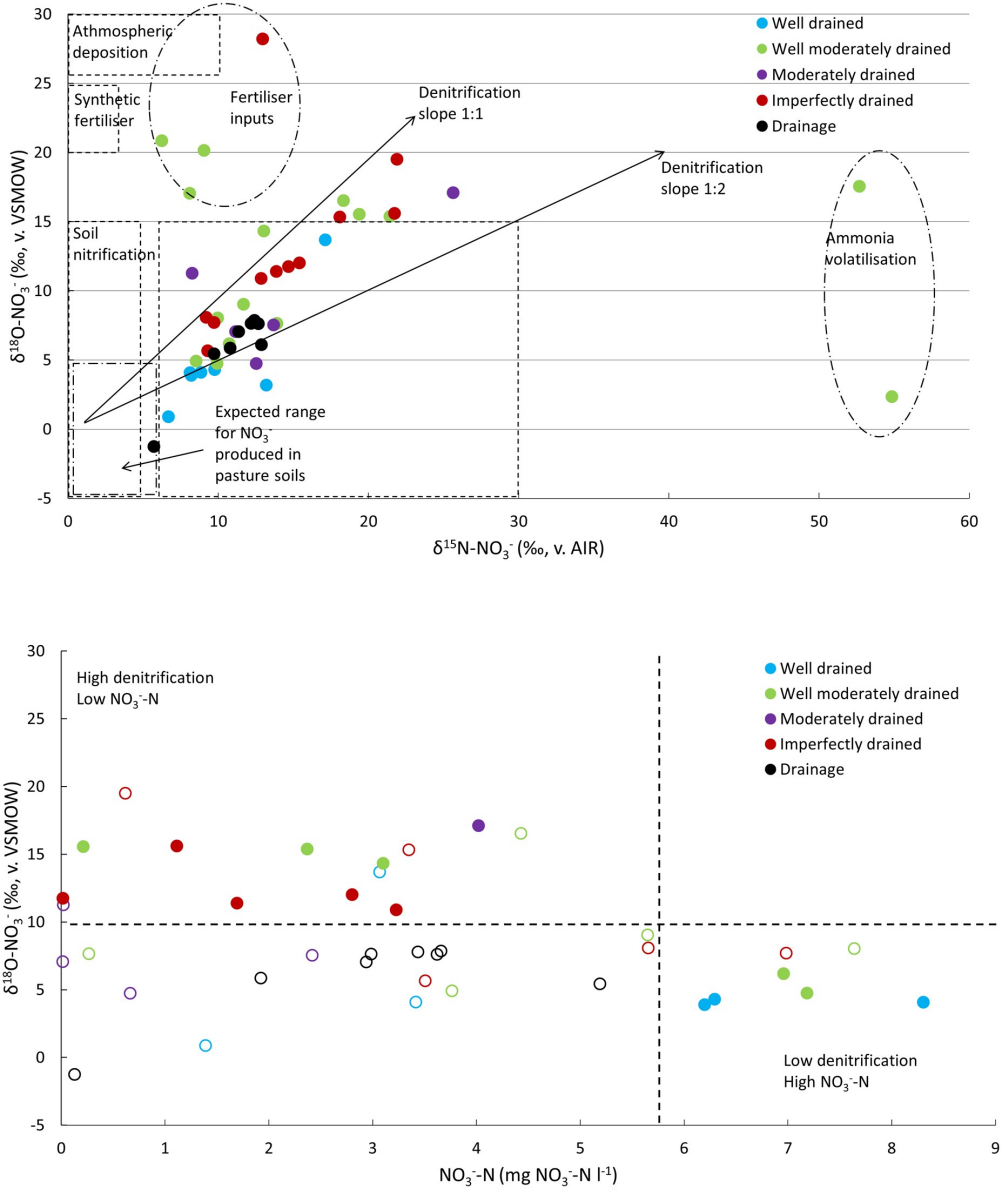
Top: scatterplot showing δ^18^O-NO_3_^-^ vs. δ^15^N-NO_3_^-^ for water samples collected in September 2014 superimposed onto δ^18^O and δ^15^N ranges for N-sources and processes by Yin et al. [49] and Baily et al. [14]. Bottom: scatterplot showing δ^18^O-NO_3_^-^ vs. NO_3_^-^-N values, identifying condition of inputs and denitrification rate. Whole circles identify wells with alternatively 1) NO_3_^-^-N concentration <5.65 mg NO_3_^-^-N l^-1^ and high denitrification isotope signature (>10‰) i.e. exhibiting excess inputs of NO_3_^-^-N that has been denitrified or 2) NO_3_^-^-N >5.65 mg NO_3_^-^-N l^-1^ and low denitrification isotopic signature (<10‰) i.e. exhibiting contamination due to an insufficient rate of denitrification. Open circles identify wells that were discarded due to depth or where the NO_3_^-^-N concentration was <5.65 mg NO_3_^-^-N l^-1^ in combination with a low denitrification isotopic signature (<10‰), i.e. exhibiting a situation of limited denitrification and low inputs.

In 2014, the average δ^15^N composition of NO_3_^-^ was 14.2‰ (6.2–54.9‰, Fig 5, S2 Table). The average δ^18^O composition of NO_3_^-^ was 9.7‰ (2.3‰-28.2‰). Isotopic values within the drainage system varied between 5.86 and 7.86‰ for δ^18^O-NO_3_^-^ and between 9.72 and 12.89 ‰ for δ^15^N-NO_3_^-^.

The up-gradient unit had isotopic values of 3.2 and 13.2‰ for δ^18^O-NO_3_^-^ and δ^15^N-NO_3_^-^, respectively. In the down-gradient unit at 0–4.5 m bgl, locations showed higher enrichment in the central and south area. This range was soil drainage class led e.g. moderate class at location 20 (17.1 and 25.6‰) and poor class at location 26 (17.6 and 52.7‰) for δ^18^O-NO_3_^-^ and δ^15^N-NO_3_^-^, respectively. The well drained area around location 21 showed enrichment only for δ^15^N-NO_3_^-^ (54.9‰). Enriched values occur between 4.5–6 m bgl at locations 24 and 25 (poor drainage) and near locations 35 and 36 (imperfect drainage). Deeper screen intervals between 6–9 m bgl, show an enrichment for δ^15^N-NO_3_^-^ in the north (11, 28.2 and 13.0‰ for δ^18^O-NO_3_^-^ and δ^15^N-NO_3_^-^) and south areas. Similar enrichments occur between 11–13 and 13–16 m bgl for both δ^18^O-NO_3_^-^ and δ^15^N-NO_3_^-^ at location 13 (19.5 and 21.9‰ for δ^18^O-NO_3_^-^ and δ^15^N-NO_3_^-^) and for δ^15^N-NO_3_^-^ at location 38 (20.9‰).

Importantly, comparing these values with [14] for the same wells, 75% of δ^15^N-NO_3_^-^ and δ^18^O-NO_3_^-^ values within 3‰ of those in 2008. In 2017, 44% and 31% of the wells were within 3‰ of those in 2008 for δ^15^N-NO_3_^-^ and δ^18^O-NO_3_^-^ respectively while 38% and 41% of the wells were within 3‰ of the 2014 values for δ^15^N-NO_3_^-^ and δ^18^O-NO_3_^-^ respectively. These three sampling events (2008, 2014 and 2017) confirm the findings of [14] that there is a consistent temporal signal.

In 2014, NO_3_^-^-N was spatially variable with some locations exceeding 5.65 mg l^-1^ (Fig 2, S2 Table). In 2008, three main areas: the north end (surrounding 5, well-drained soil), with a value of 9.5 mg NO_3_^-^-N l^-1^ (±2.9); the central area (surrounding 14, well-drained soil), with a value of 10.3 mg NO_3_^-^-N l^-1^ (±4.5); and south end (surrounding 28, well- to moderately- drained soil), with a value of 7.3 mg NO_3_^-^-N l^-1^ (±2.4) [14] were elevated. High NO_3_^-^-N was attributed to old DSW irrigation areas and farmyard leachate (see [14]). In Sep 2014 NO_3_^-^-N < 5.65 was evident for all locations (Fig 5). In terms of N source groundwater NO_3_^-^-N isotopic composition clustered within the manure/sewage value range and along a 1:1–1:2 slope, suggesting a common organic source for these, and denitrification as the main biotransformation process (Fig 5) [41–43]. The denitrification lines for 2014 and 2017 samples share the same trend, which points to denitrification as the main process leading N attenuation. Distinguishing between denitrification and DNRA is difficult as the isotope effect of DNRA has still not been investigated [44,45].

The use of a modified Rayleigh equation to estimate NO_3_^-^-N attenuation [46] is acceptable as the source on site is uniform and the source of water on site has been established. Values between -3 to -30‰ have been reported for ε_denit_ [42,47]. Herein, the ratio of δ^18^O:δ^15^N enrichment during denitrification remained constant. Therefore, enrichment in both δ^15^N-NO_3_^-^ and δ^18^O-NO_3_^-^ can be attributed to biotransformation of NO_3_^-^-N and is directly proportional to the degree of denitrification (*f*) [45,46]. Across the entire site, only a few wells showed high temporal variability, possibly due to management, compared with values in 2008 and 2014–2017. Such wells showed alternatively high values of δ^18^O-NO_3_^-^ or δ^15^N-NO_3_^-^ and exhibited a shift from the 1:1–1:2 slope (Fig 5) [48]. From this group two other signatures emerged: a high enrichment of δ^15^N-NO_3_^-^ possibly from surface NH_3_ volatilization, and high δ^18^O-NO_3_^-^ values, possibly due to an atmospheric source or synthetic fertilizer as nitrate source (Fig 5).

The subset of data points, close to the 1:1 isotope ratio line, where denitrification was the dominant process, was further examined by eliminating points with a fertiliser and ammonia volatilisation signature (Fig 5). A plot of δ^18^O-NO_3_^-^ versus NO_3_^-^-N revealed three main groups, each identifying a specific condition of inputs and extent of denitrification (Fig 5). The first group had a low NO_3_^-^-N concentration (<5.65 mg NO_3_^-^-N l^-1^) associated with limited denitrification isotopic signature (<10‰) and low inputs. The second group had a low NO_3_^-^-N concentration but high denitrification isotope signature (>10‰), indicating that excess inputs of NO_3_^-^-N have been denitrified. The final group had high NO_3_^-^-N (>5.65 mg NO_3_^-^-N l^-1^) and low denitrification isotopic signature (<10‰), highlighting contamination but limited denitrification. The first group was discarded from analysis as not influential in terms of denitrification and contamination, while groups with NO_3_^-^-N above 5.65 mg NO_3_^-^-N l^-1^ or δ^18^O-NO_3_^-^ above 10‰ were selected. Poorly-drained soils are characterised by high denitrification potential and low NO_3_^-^-N, whereas well-drained soils had low denitrification potential and higher NO_3_^-^-N. These isotopic enrichment patterns followed soil permeability patterns which were consistent with the findings of Fenton et al. [13] (Fig 5).

Natural isotopic abundance for NH_4_^+^-N was measured in wells with detectable NH_4_^+^-N concentration. The average δ^15^N-NH_4_^+^ farm concentration was 18.5‰ (max 34.3‰ corresponded with breach of MAC; min 3.3‰). The site average N_2_O stable isotope composition was 14.2‰ for δ^18^O-N_2_O and -17.1‰ for δ^15^N-N_2_O, respectively. Maximum values were 46.7‰ and 21.4‰ for δ^18^O-N_2_O and δ^15^N-N_2_O, respectively, while minimum values were -14.5‰ and -32.5‰. NH_4_^+^-N polluted wells occur mainly in the central area of the farm (Fig 3) with moderate- to imperfectly-drained soil. A high NH_4_^+^-N concentration was evident in wells showing relative enrichment in δ^18^O-NO_3_^-^ and with a synthetic fertilizer source signature (Fig 5). However, a high NH_4_^+^-N concentration could occur without this signature, due to *in-situ* transformation of NO_3_^-^ to NH_4_^+^ by DNRA [11,15]. Even though denitrification and DNRA occur under similar environmental conditions, DNRA has rarely been observed with respect to denitrification but is an important process under anaerobic conditions with high (>12) C/NO_3_^-^ ratio [49,50]. High NH_4_^+^-N location showed lower (p<0.05) dissolved oxygen concentration (av. 0.65 mg l^-1^) and NO_3_^-^-N (av. 0.94 mg NO_3_^-^-N l^-1^) than low NH_4_^+^-N locations (av. 3.72 mg l^-1^ and 3.46 respectively mg NO_3_^-^-N l^-1^). No significant differences between high and low NH_4_^+^-N location were found in terms of DOC concentrations or groundwater level. Since no direct fractionation factors exist for DNRA [51], δ^15^N-NH_4_^+^ values were measured to assess a possible role of DNRA. As per Rayleigh fractionation, we hypothesised that DNRA would lead to a δ^15^N-NH_4_^+^ value significantly less enriched than at a location where denitrification was the dominant process. With only four locations having a C/NO_3_^-^ ratio >12, δ^15^N-NH_4_^+^ signatures did not show any distinct pattern. This could indicate that DNRA was of secondary importance and restricted to a few locations at high depth or micropores within the soil profile (S5 Fig).

### Conceptual diagram of the site to inform soil nitrogen attenuation potential

**Fig 6:**
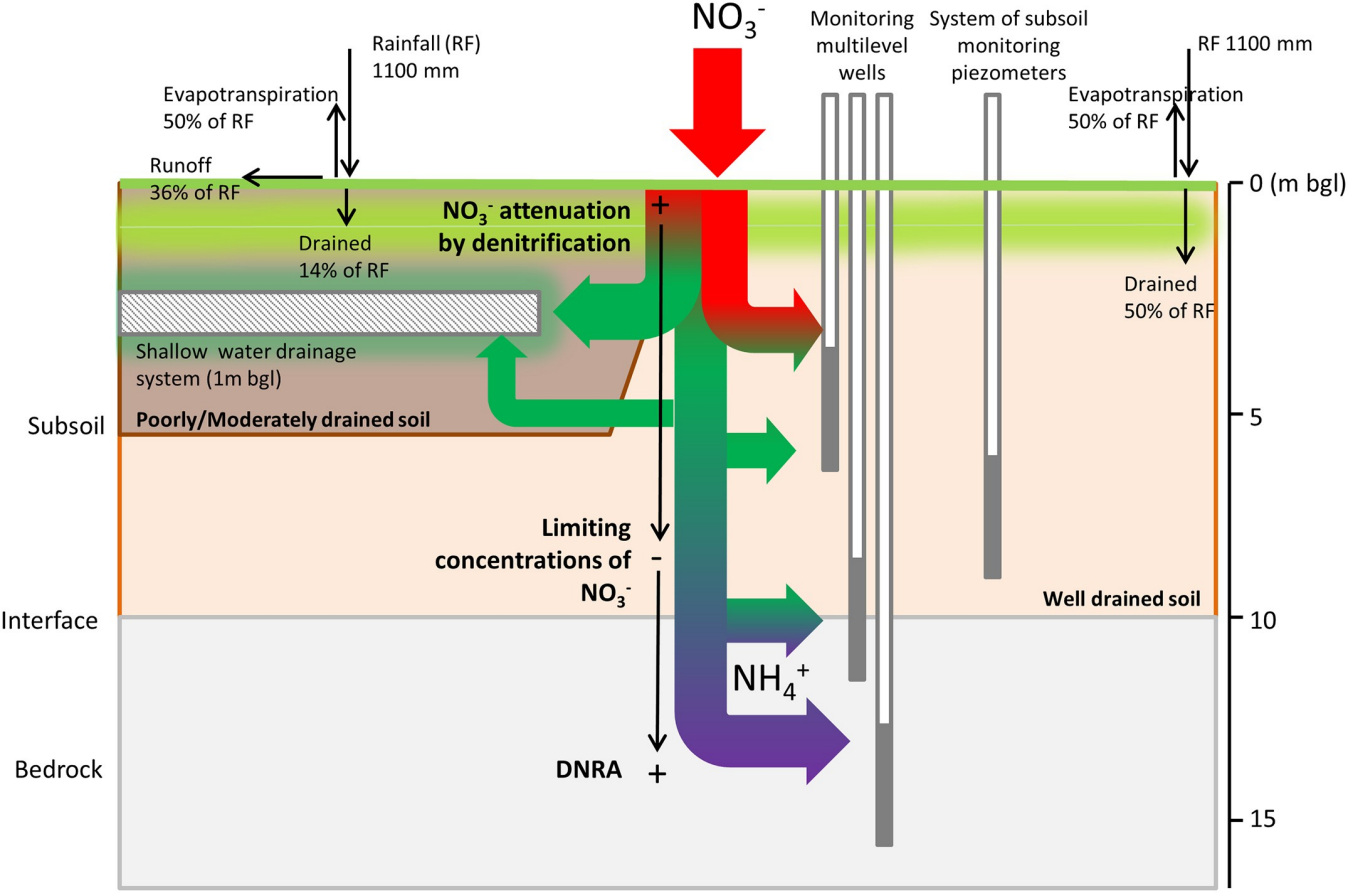
Conceptual diagram of the two-tiered system beneath the site: 1) a shallow migration pathway in poorly-imperfectly drained soils with high N_O_^3^-N attenuation which is not disrupted by the artificial drainage system to the outlet; 2) a deep migration pathway under moderately-well drained conditions where N_O_^3^-N attenuation is lower therefore leading to its transformation in NH_4_^+^-N.

Integrating all data (Fig 6), four main location groups were distinguished with respect to potential for soil N attenuation (Fig 1, S2 Table):

*Group 1 (high N attenuation potential)*: locations with NH_4_^+^-N and NO_3_^-^-N concentrations below MAC. Drainage class of these soils are imperfectly to moderately-well drained and drainage water from these areas does not represent a threat to water quality. This condition reflects relatively high NO_3_^-^-N attenuation rates, via denitrification. Generally low N_2_O emissions inferred completeness of denitrification, although some locally elevated N_2_O production suggested concurrent secondary processes (e.g. nitrification), with a possible threat to air quality.

*Group 2 (moderate N attenuation potential)*: locations with a NO_3_^-^-N concentration below the contamination threshold, but with high N_2_O production. Drainage classes are well to moderately-drained and drainage water from these soils does not represent a threat to water quality, but instead is a problem in terms of GHG emissions. Such emissions are due to a low capacity for denitrification, resulting in low NO_3_^-^-N, possibly coupled with additional N_2_O emissions from nitrification.

*Group 3a (low N attenuation potential*, *DSW irrigation*): locations with a NO_3_^-^-N concentration above significant contamination. Drainage classes are mainly well to moderately-well drained, with a higher permeability than those in Group 1 and drainage water from these locations represents water and air quality issues (due to high N_2_O emissions, which indicate incomplete denitrification). This results from a medium-low potential for NO_3_^-^-N attenuation by denitrification and if drained will present a threat to groundwater and GHG emissions.

*Group 3b (low N attenuation potential*): locations with low NO_3_^-^-N but NH_4_^+^-N above NH_4_^+^-N MAC and high N_2_O emissions. Drainage classes are well to imperfectly-drained with generally low potential for denitrification and drainage water is a threat to groundwater and GHG emissions.

De Klein et al. [52] observed that more than 50% of EU dairy farms have high N surpluses (>200 kg N ha^-1^), with only 7% showing values below 100 kg N ha^-1^. The N surplus on our Irish farm was high and above the national average of 175 kg N ha^-1^ [53]. As a direct continuum has been shown to exist N can transported and transformed as follows (Fig 6):

1. a shallow migration pathway in poorly- or imperfectly-drained soils with high water purification capability. The drainage system does not disrupt this capacity but instead conveys clean water to the exit point of the farm;
2. a deep migration pathway in moderately- and well-drained soils where the water purification capability is lower. This facilitates leaching of N, which is then converted at depth to NH_4_^+^-N and migrates off site along deeper groundwater pathways.

### Development of farm management strategy for nitrogen

The farm is operating within the current regulations [54–56], following the EU Nitrates Directive [57], but these assume homogeneous soil-subsoil-geology without variation in N natural attenuation capacity. The present study shows that four attenuation capacity areas exist, with Group 3a and 3b areas representing the most vulnerable. N loads need to be diverted away from these areas e.g. DSW produced needs to be reduced and irrigation on the farm should avoid group 3a and b areas, and be reduced on group 2 areas.

Historically, the DSW irrigation system has been moved around (e.g. after a long period in 2005 it was moved from heavier soils along the central drainage ditch to avoid overland flow losses) and since then it is positioned behind the farmyard on well- to moderately-drained soils. This static position has concentrated a high N load in this area and can be correlated with elevated NO_3_^-^-N in down-gradient groundwater. Presently all DSW is collected in a three-chamber separation farmyard underground tank (total capacity ~55,000 l) with a variable retention time depending on volume of washings and rainfall. In periods where land is saturated and spreading with Roto-Rainer is not possible, DSW can be stored in an earthen bank lagoon. This DSW is spread with an umbilical cord system within the downstream farm on the three main sections below the built-up area in Fig 1 (plots with locations 28, 33 and 35).

Farmyard measures must be optimised to manage the volume of DSW produced on the farm, through 1) Reduction of DSW production—Diversion of all clean water to a clean water outfall, preventing clean water from becoming soiled and restructuring of the yard to minimise DSW generation; 2) Optimisation of DSW spreading—Use of the information gained from the present study (heterogeneous soil functionalities) to better use the entire farm to spread DSW. The following management options are considered to be viable in terms of reducing the N load:

1. Short term management options: 1) Moving DSW irrigation to high N attenuation areas (Group 1 imperfectly- to moderately-well drained soil locations), or moderate N attenuation areas (Group 2 well- to moderately-drained soil locations), where no overland flow component could affect the open ditch network; 2) Removal of the N load from vulnerable areas that will allow the N biogeochemical time lag to diminish over time, which should result in a decreased groundwater NO_3_^-^-N concentration.
2. Long term management option: minimise the N source whilst using the lagoon for storage. This provides flexibility for DSW to be 1) land spread when its fertilizer replacement value is high, or 2) discharged to a constructed wetland installed at the end of the farm (Group 1 location) at other times, e.g. over winter. The lagoon concept has been considered on dairy farms [58,59]. The loss of land for the lagoon would not be severe as full utilisation of the area currently being used for DSW application is difficult at times due to ground trafficability and grass palatability. In addition, the labour lost through the management of an irrigation system would be gained for other activities on the farm. Significant energy saving is also achievable with the significant reduction in pumping of DSW. One consideration here is to design the system for expansion in the event that the discharge licence is not met over time, in which case a de-sludging specification should be considered.

## Conclusions

Integrating surface and sub-surface spatial and temporal data on an intensive dairy farm provided knowledge on N load and surplus, N source-transformation and fate along surface and subsurface pathways. This information enabled the dairy farm to be divided into four distinct areas based on N attenuation capacity. This method has the ability to match future N loads with attenuation capacity, thereby optimising the farm system in terms of environmental sustainability.

## Supporting information

S1 Fig NO_3_^-^-N, NH_4_^+^-N and NO_2_^-^-N variation across the farm from Dec 2005 to Dec 2017. (TIF)

S2 Fig Depth specific excess-N_2_ concentration on the farm collected on Sep 2014. Top left: drainage system, top right: 2.95–4.5 m bgl, middle left 4.5–6 m bgl, middle right 6–9 m bgl, bottom left 11–13 m bgl, bottom right: below 16 m bgl.
(TIF)

S3 Fig Depth specific EF5g (1) concentration on the farm collected on Sep 2014. Top left: drainage system, top right: 2.95–4.5 m bgl, middle left 4.5–6 m bgl, middle right 6–9 m bgl, bottom left 11–13 m bgl, bottom right: below 16 m bgl.
(TIF)

S4 Fig δ^18^O versus δ^15^N-N_2_O values for samples collected in Sep 2014. Red lines represent the limits for N_2_O production calculated for the farm (JC site). Black squares represent source as delineated by (Li et al., 2014).
(TIF)

S5 Fig NH_4_^+^-N concentration vs. δ^15^N-NH_4_^+^ values for samples collected in Sep 2014. (TIF)

S1 Table Data sources used in addition to the present fieldwork. Nutrient concentrations: nitrate-N concentration (NO_3_^-^ -N), nitrite-N concentration (NO_2_^-^ -N), ammonium-N concentration (NH_4_^+^ -N), total nitrogen (TN), total organic nitrogen (TON), phosphorus (PO_4_^3-^), total phosphorus (TP), dissolved reactive phosphorus (DRP). Physiochemistry: dissolved oxygen (DO), electrical conductivity (EC), redox potential (Eh), pH, calcium (Ca^2+^), chloride (Cl^-^), copper (Cu^2+^), potassium (K^+^), iron (Fe^2+^), manganese (Mn^2+^), magnesium (Mg^2+^), sodium (Na^-^), sulphide (S^2-^), sulphate (SO_4_^+^), zinc (Zn^2+^), dissolved organic carbon (DOC). Isotope: δ^18^O-NO_3_^-^ values, δ^15^N-NO_3_^-^ values. Dissolved gasses: nitrous oxide (N_2_O), molecular nitrogen (N_2_), carbon dioxide (CO_2_), methane (CH_4_). Others: Water table (WT), vertical travel time (Tt), Effective rainfall (ER), effective drainage (ED), potential evapotranspiration (PET), actual evapotranspiration (AET), soil moisture deficit (SMD), saturated hydraulic conductivity (k_s_).
(DOCX)

S2 Table Sustainability groups, depth, concentrations of N species (NO_3_^-^-N, NH_4_^+^-N, NO_2_^-^-N, dissolved N_2_O, excess-N_2_) and signatures (δ^15^N-NO_3_^-^δ^18^O-NO_3_^-^) variation across the locations in Sept 2014. (DOCX)
