# Supplementary figures and images for: An integrated assessment of nitrogen source, transformation and fate within an intensive dairy system to inform management change
Source: PLoS One. 2019 Jul 23;14(7):e0219479. doi: 10.1371/journal.pone.0219479 (PMC6650055; doi:10.1371/journal.pone.0219479)

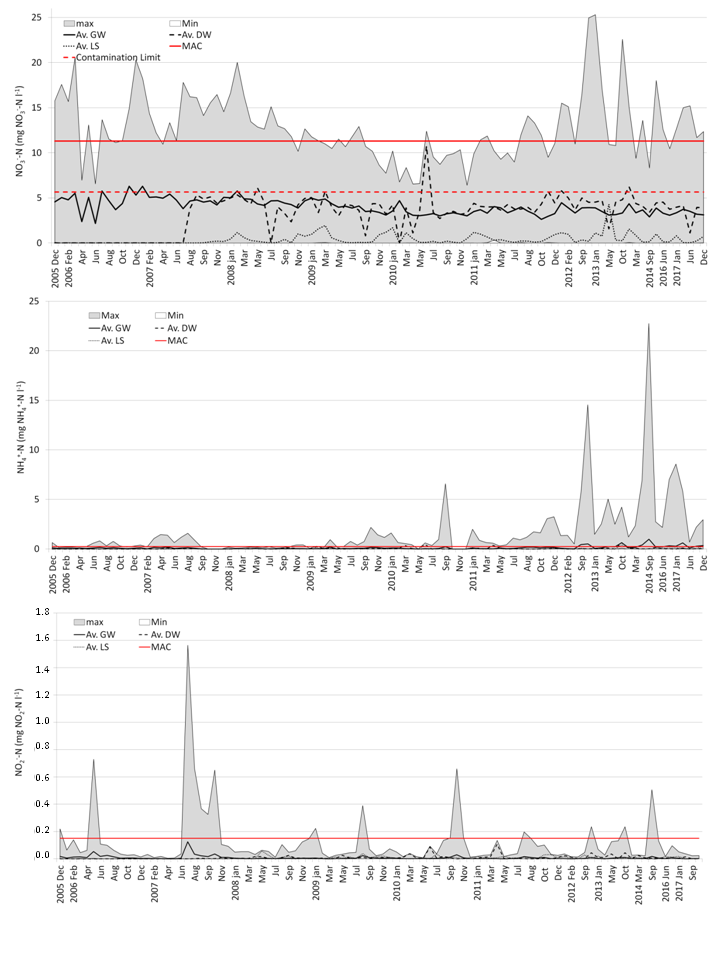

Supplement: S1 Fig — (TIF) [file pone.0219479.s001.tif]

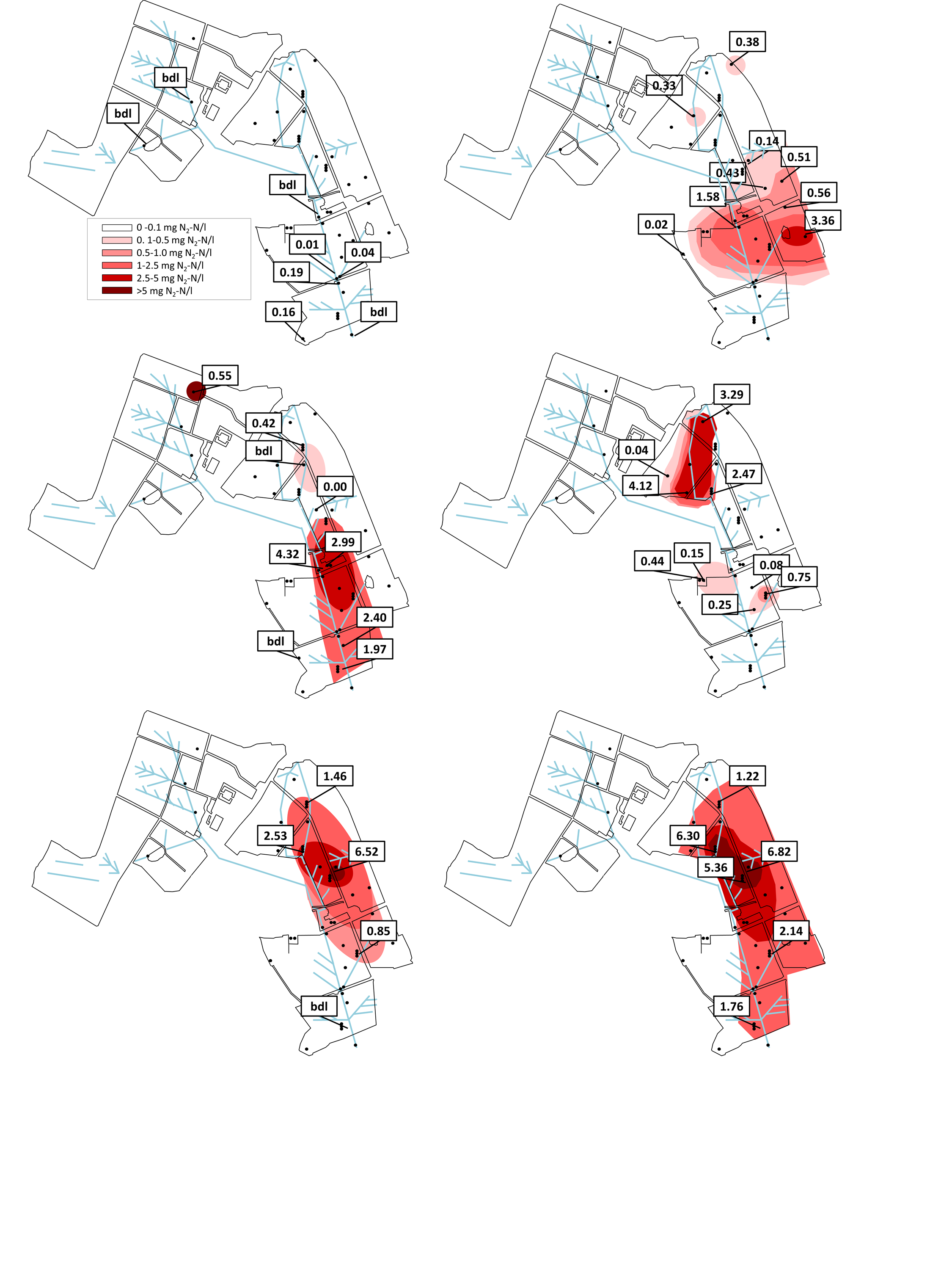

Supplement: S2 Fig — Top left: drainage system, top right: 2.95–4.5 m bgl, middle left 4.5–6 m bgl, middle right 6–9 m bgl, bottom left 11–13 m bgl, bottom right: below 16 m bgl. (TIF) [file pone.0219479.s002.tif]

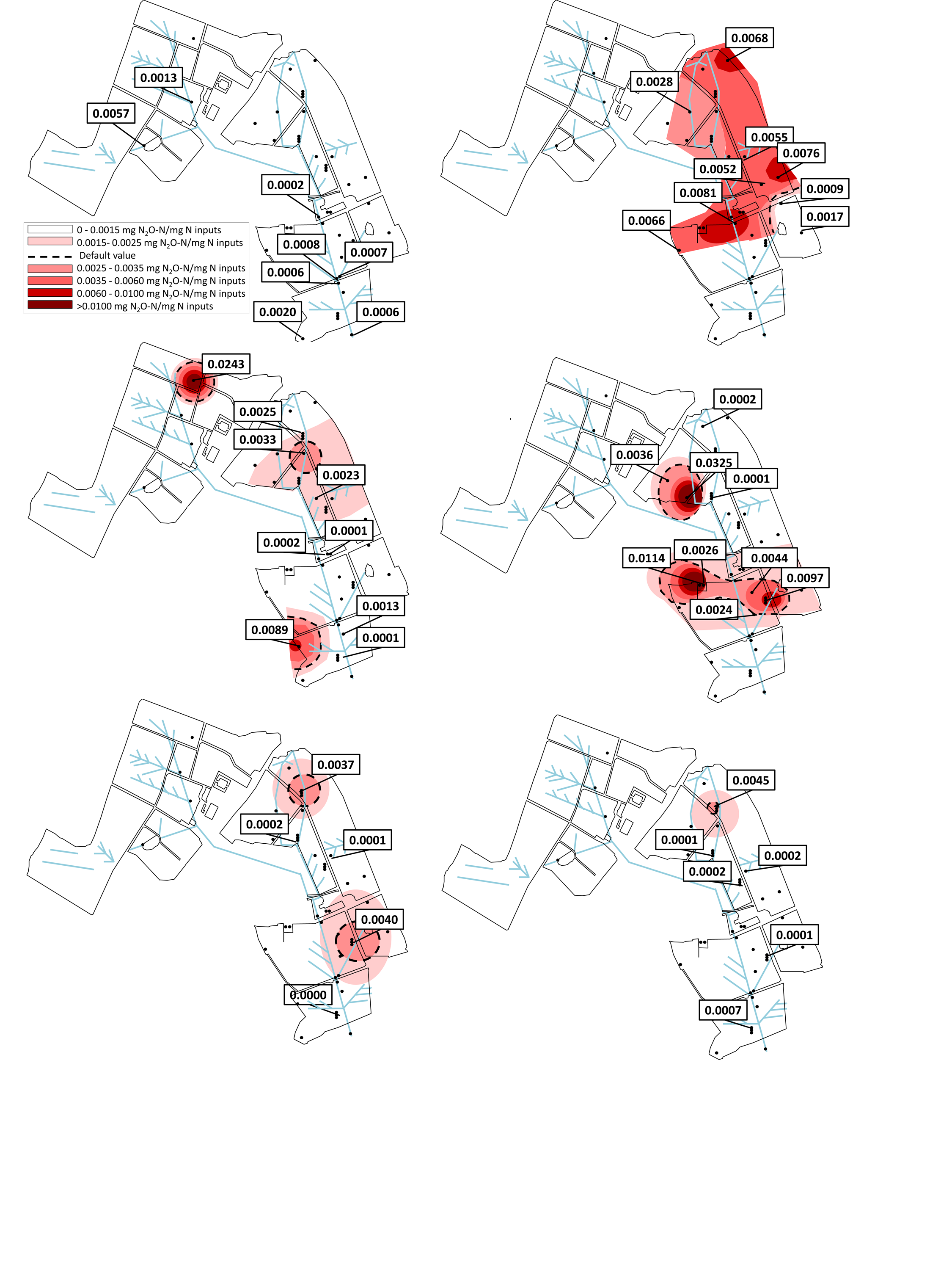

Supplement: S3 Fig — Top left: drainage system, top right: 2.95–4.5 m bgl, middle left 4.5–6 m bgl, middle right 6–9 m bgl, bottom left 11–13 m bgl, bottom right: below 16 m bgl. (TIF) [file pone.0219479.s003.tif]

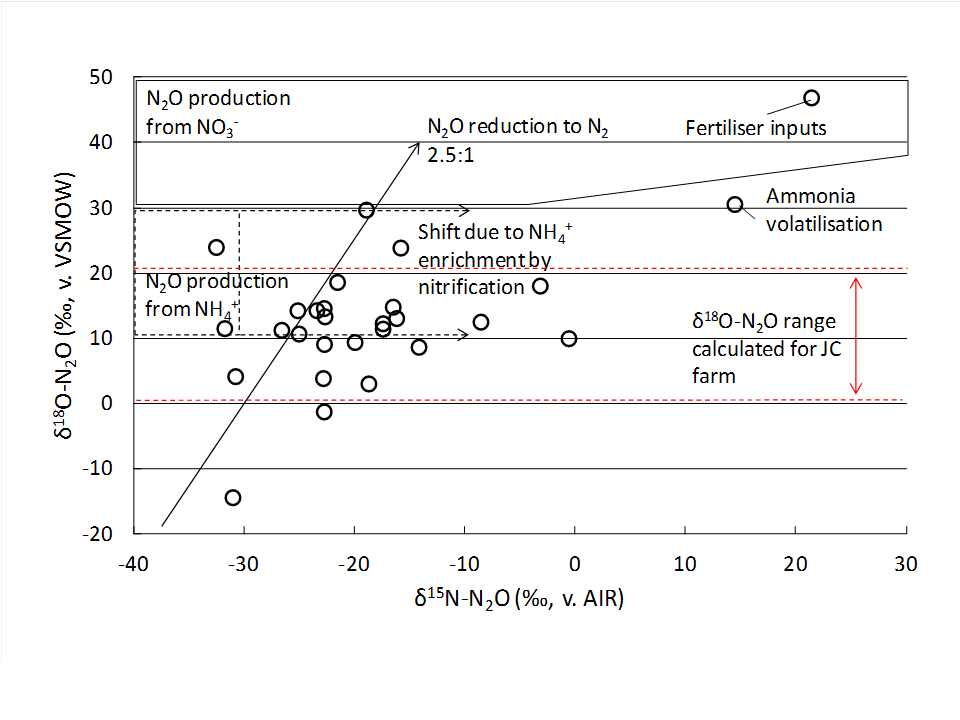

Supplement: S4 Fig — Red lines represent the limits for N2O production calculated for the farm (JC site). Black squares represent source as delineated by (Li et al., 2014). (TIF) [file pone.0219479.s004.tif]

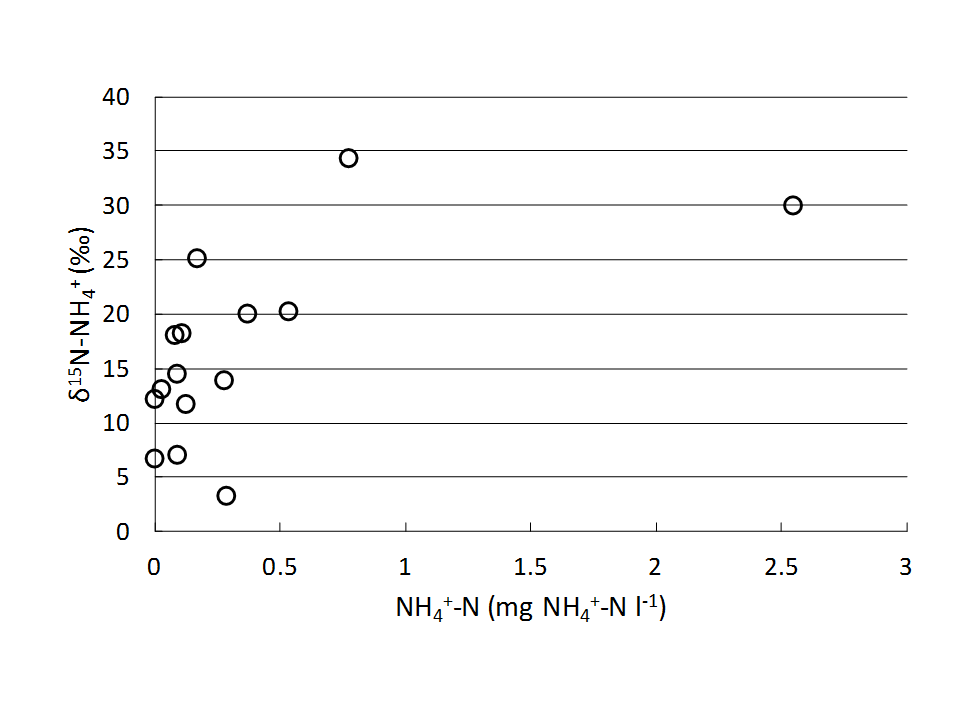

Supplement: S5 Fig — (TIF) [file pone.0219479.s005.tif]
