# Supplementary material for: An integrated assessment of nitrogen source, transformation and fate within an intensive dairy system to inform management change
Source: PLoS One. 2019 Jul 23;14(7):e0219479. doi: 10.1371/journal.pone.0219479 (PMC6650055; doi:10.1371/journal.pone.0219479)
Supplement: S1 Table — Nutrient concentrations: nitrate-N concentration (NO3- -N), nitrite-N concentration (NO2- -N), ammonium-N concentration (NH4+ -N), total nitrogen (TN), total organic nitrogen (TON), phosphorus (PO43-), total phosphorus (TP), dissolved reactive phosphorus (DRP). Physiochemistry: dissolved oxygen (DO), electrical conductivity (EC), redox potential (Eh), pH, calcium (Ca2+), chloride (Cl-), copper (Cu2+), potassium (K+), iron (Fe2+), manganese (Mn2+), magnesium (Mg2+), sodium (Na-), sulphide (S2-), sulphate (SO4+), zinc (Zn2+), dissolved organic carbon (DOC). Isotope: δ18O-NO3- values, δ15N-NO3- values. Dissolved gasses: nitrous oxide (N2O), molecular nitrogen (N2), carbon dioxide (CO2), methane (CH4). Others: Water table (WT), vertical travel time (Tt), Effective rainfall (ER), effective drainage (ED), potential evapotranspiration (PET), actual evapotranspiration (AET), soil moisture deficit (SMD), saturated hydraulic conductivity (ks). (DOCX) [file pone.0219479.s006.docx]

**S1 Table.**

| **Source** | **Data collected** | **Approach** | **Contribution to present work** | **Times and locations** |
| --- | --- | --- | --- | --- |
| 1. Met station | - Daily Tmax, Tmin, total rainfall, main wind speed, solar radiation | Download | Annual Rainfall and national weather condition, used in conjunction with soil drainage class to elucidate ED | Jan. 2008 – Nov.2014  Downstream unit |
| 1. Fertiliser dataset   (unpublished  data) | - Inputs: N, K and P (urea, CAN, farmyard manure, dirty water, slurry, woodchip, MOP, Super phosphate) | Farm records | Fertiliser inputs, types, locations of yards and storage facilities for DSW | Jan. 2007 – Oct. 2014  Both units |
| 1. Johnstown Castle Soil Map | - Soil type (1-20 cm bgl) - Soil drainage class - Depth to bedrock | Map and report available | Soil type with associated drainage class; Indicative permeability | Both units |
| 1. Kurz et al., [60] | - Nutrients (NH_4_^+^-N, TON, DRP, K^+^). - Fertilizers use (N, P, K). - Runoff | Field work | Nutrient concentration at limited locations along the old sub-surface piped drainage system; Correlation between management and water chemistry in drainage sections; Proportion of overland flow vs. drainage flow | Nov. 1996 - May 1997  Some sections of both units |
| 1. Groundwater quality dataset   (unpublished data) | - GWT (Dec. 2005 - Jun. 2014). - pH, T, EC, Turb., DO and Eh (Mar. 2009- Jun. 2014). - Ca^2+^, Mg^2+^, K^+^, DRP, TP and Na^-^ (Dec. 2005 - Sep. 2010). - Cu^2+^, Fe^2+^, Mn^2+^ and Zn^2+^ (Dec. 2008 - Sep. 2010). - Cl^-^, NO_3_^-^ -N, NO_2_^-^-N, NH_4_^+^-N, TN, DON and DOC (Dec. 2005-Jun. 2014). | Grab water samples collection (manually, peristaltic, bladder pump),  In situ probe and  Physiochemical analyses | Spatial and temporal distribution of water quality parameter | Dec. 2005 – Jun. 2017 (monthly, Dec. 2005 – Dec. 2011, bimonthly, Jan. 2012 – Jun. 2017)  Dairy farm: 2 multilevel boreholes (11-13; 36, 37, 38), 17 shallow piezometer (2, 3, 4, 5, 6, 10, 19, 20, 21, 26, 27, 28, 29, 33, 34, 35) 3 boreholes (18, 24, 25) and 4 surface locations D4, D7, D8, D9) (n. 2, 11, 12, 13, 19, 20, 21, 24, 28, 36, 37, 38, Dec. 2005 – Jun. 2014; n. 3, 4, 5, 6, 10, 26, 27, 29, 33, 34, 35, Jul. 2007 – Jun. 2014, D4, D7, D8 May 2007 – Jun. 2014, D9 Jul. 2007 – Jun. 2014) |
| 1. Baily et al., [14] | - Nutrients (NO_3_^-^ -N). - Hydrochemistry (Tt, Cl^-^, DO). - Isotopes (δ^18^O-NO_3_^-^, δ^15^N-NO_3_^-^ ). - GWT, k_s_. | Natural isotopic abundance | Correlates nitrates with a source e.g. organic fertilizer; Transformational processes; location of hotspots  Vertical travel time to shallow groundwater in site varied from months to years. | Apr., Aug., Dec. 2008  Dairy farm, shallow piezometer network (L3, L5, L8, L9, L11, L13, L14, L16, L17, L18, H1, H2, H5, H6, H9, A1) |
| 1. Jahangir et al., [10] | - Nutrients (NO_3_^-^-N, TN, DON). - Hydrochemistry (DOC, TC). - Dissolved gasses (N_2_O, N_2_, CO_2_, CH_4_). - GWT, ER, PET, AET, k_s_. | Physiochemical and gaseous analyses | Farm N-balance with surplus; Quantification of farm scale indirect GHG emissions; Role of site characteristics in the partitioning of N losses | Feb. 2009 – Jan. 2011 (monthly)  Dairy farm, multi-level boreholes (7, 8, 9, 11, 12, 13, 15, 16, 17, 22, 24, 18, 25, 30, 31, 32, 36, 37, 38) |
| 1. Jahangir et al., [11] | - Nutrients (NO_3_^-^-N, NO_2_^-^-N, NH_4_^+^-N, TN). - Physiochemistry (DO, ORP, pH, Cl^-^, Fe^2+^, Mn^2+^, S^2-^, SO_4_^+^, DOC). - Dissolved gasses (CO_2_, CH_4_). - GWT, SMD, ER, PET, AET, k_s_. | Physiochemical and gaseous analyses | Soil type and bedrock geology; Physiochemical variations and correlation with denitrification rates; Localisation of hot spot due to waste water irrigation practices | Feb. 2009 – Jan. 2011 (monthly)  Dairy farm, multi-level boreholes (7, 8, 9, 11, 12, 13, 15, 16, 17, 22, 24, 18, 25, 30, 31, 32, 36, 37, 38) |
| 1. Jahangir et al., [15] | - Nutrients (NO_3_^-^ -N, NO_2_^-^ -N, NH_4_^+^ -N, TN, PO_4_^3-^). - Physiochemistry (DO, ORP, pH, Cl^-^, Fe^2+^, Mn^2+^, S^2-^, SO_4_^+^, DOC). - Dissolved gasses (N_2_O, N_2_). - GWT, SMD, ER, PET, AET, k_s_. | Physiochemical and gaseous analyses | Farm scale N balance with surplus; Indirect gaseous emissions trends according to hydrology and depth | Feb. 2009 – Jan. 2011 (monthly)  Dairy farm, multi-level boreholes (7, 8, 9, 11, 12, 13, 15, 16, 17, 22, 24, 18, 25, 30, 31, 32, 36, 37, 38) |
| 1. Jahangir et al., [21] | - NO_3_^-^ -N in groundwater - Physiochemistry (DO, ORP, pH, Fe^2+^, S^2-^, SO_4_^+^, DOC).   - Dissolved gasses (N_2_O, N_2_).  - GWT, SMD, ER, PET, AET, k_s_ | Push and Pull | Soil; Groundwater denitrification rates | Oct., Dec. 2010  Dairy farm, multi-level boreholes (7, 8, 9, 15, 16, 17, 30, 31, 32) |

Supplementary References

60. Kurz, I., Coxon, C., Tunney, H., Ryan, D., 2005. Effects of grassland management practices and environmental conditions on nutrient concentrations in overland flow. Journal of Hydrology, 304 (1-4), 35-50.
