# Supplementary material for: An integrated assessment of nitrogen source, transformation and fate within an intensive dairy system to inform management change
Source: PLoS One. 2019 Jul 23;14(7):e0219479. doi: 10.1371/journal.pone.0219479 (PMC6650055; doi:10.1371/journal.pone.0219479)
Supplement: S2 Table — (DOCX) [file pone.0219479.s007.docx]

**S2 Table.**

| Wells | Depth | NO_3_^-^-N | NO_4_^+^-N | NO_2_^-^-N | Dissolved N_2_O | Excess-N_2_ | δ^15^N-NO_3_^-^ | δ^18^O-NO_3_^-^ |
| --- | --- | --- | --- | --- | --- | --- | --- | --- |
|  | (m bgl) | (mg NO_3_^-^-N l^-1^) | (mg NO_4_^+^-N l^-1^) | (mg NO_2_^-^-N l^-1^) | (mg N_2_O-N l^-1^) | (mg N_2_-N l^-1^) | (‰) | (‰) |
| Group 1 |  |  |  |  |  |  |  |  |
| 7 | 2.95 - 4.5 | 2.80 | 0.00 | 0.06 | 0.0087 | 0.42 | 15.4 | 12.0 |
| 15 | 2.95 - 4.5 | 0.66 | 0.00 | 0.00 | 0.0097 | 0.14 | 12.5 | 4.8 |
| 21 | 2.95 - 4.5 | 0.06 | 0.00 | 0.00 | 0.0007 | 0.56 | 54.9 | 2.4 |
| 25 | 4.5 - 6 | 2.37 | 0.00 | 0.00 | 0.0004 | 2.99 | 21.4 | 15.4 |
| 34 | 4.5 - 6 | 0.27 | 0.00 | 0.00 | 0.0033 | 0.00 | 13.9 | 7.7 |
| 35 | 4.5 - 6 | 1.11 | 0.08 | 0.00 | 0.0050 | 2.40 | 21.8 | 15.6 |
| 36 | 4.5 - 6 | 0.21 | 0.09 | 0.00 | 0.0002 | 1.97 | 19.4 | 15.5 |
| 18 | >16 | 3.07 | 0.00 | 0.00 | 0.0017 | 5.36 | 17.1 | 13.7 |
| 32 | >16 | 4.43 | 0.00 | 0.00 | 0.0005 | 2.14 | 18.3 | 16.5 |
| 38 | >16 | 0.00 | 0.09 | 0.00 | 0.0014 | 1.76 | 6.2 | 20.9 |
| Average |  | 1.50 | 0.03 | 0.01 | 0.0032 | 1.77 | 20.1 | 12.4 |
| Group 2 |  |  |  |  |  |  |  |  |
| 3 | 2.95 - 4.5 | 1.69 | 0.00 | 0.00 | 0.0165 | 0.38 | 13.9 | 11.4 |
| 19 | 2.95 - 4.5 | 2.42 | 0.00 | 0.00 | 0.0188 | 0.43 | 13.7 | 7.5 |
| 20 | 2.95 - 4.5 | 4.02 | 0.02 | 0.00 | 0.0390 | 0.51 | 25.6 | 17.1 |
| 26 | 2.95 - 4.5 | 0.30 | 0.12 | 0.05 | 0.0192 | 1.58 | 52.7 | 17.6 |
| 33 | 2.95 - 4.5 | 3.10 | 0.08 | 0.00 | 0.0120 | 3.36 | 13.0 | 14.3 |
| 1 | 4.5 - 6 | 3.44 |  |  | 0.1186 | 0.55 | 13.2 | 3.2 |
| 10 | 4.5 - 6 | 3.23 | 0.00 | 0.00 | 0.0120 | 0.00 | 12.9 | 10.9 |
| 22 | 6 - 9 | 1.39 | 0.00 | 0.04 | 0.0224 | 0.44 | 6.7 | 0.9 |
| 23 | 6 - 9 | 3.42 | 0.17 | 0.00 | 0.0111 | 0.15 | 8.9 | 4.1 |
| 29 | 6 - 9 | 3.76 | 0.00 | 0.00 | 0.0108 | 0.25 | 8.5 | 4.9 |
| 30 | 6 - 9 | 5.65 | 0.00 | 0.00 | 0.0678 | 0.75 | 11.7 | 9.0 |
| Average |  | 2.95 | 0.04 | 0.01 | 0.0317 | 0.76 | 16.4 | 9.2 |
| Group 3a |  |  |  |  |  |  |  |  |
| 5 | 2.95 - 4.5 | 8.31 | 0.00 | 0.00 | 0.0263 | 0.33 | 8.1 | 4.1 |
| 27 | 2.95 - 4.5 | 7.19 | 0.00 | 0.00 | 0.0526 | 0.02 | 9.9 | 4.7 |
| 14 | 4.5 - 6 | 6.30 | 0.00 | 0.00 | 0.0164 | 0.00 | 9.8 | 4.3 |
| 4 | 6 - 9 | 6.20 | 0.11 | 0.00 | 0.0244 | 0.04 | 8.2 | 3.9 |
| 28 | 6 - 9 | 6.96 | 0.03 | 0.00 | 0.0332 | 0.08 | 10.7 | 6.2 |
| 8 | 11 - 13 | 6.98 | 0.00 | 0.00 | 0.0324 | 1.46 | 9.7 | 7.7 |
| 31 | 11 - 13 | 7.64 | 0.00 | 0.00 | 0.0364 | 0.85 | 10.0 | 8.0 |
| 9 | >16 | 5.65 | 0.00 | 0.00 | 0.0333 | 1.22 | 9.2 | 8.1 |
| Average |  | 6.90 | 0.02 | 0.00 | 0.0319 | 0.50 | 9.5 | 5.9 |
| Group 3b |  |  |  |  |  |  |  |  |
| 24 | 4.5 - 6 | 0.04 | 0.77 | 0.00 | 0.0009 | 4.32 | 8.1 | 17.0 |
| 6 | 6 - 9 | 3.51 | 2.55 | 0.51 | 0.3588 | 4.12 | 9.3 | 5.7 |
| 12 | 11 - 13 | 3.35 | 0.28 | 0.00 | 0.0016 | 2.53 | 18.1 | 15.3 |
| 16 | 11 - 13 | 0.02 | 0.29 | 0.00 | 0.0008 | 6.52 | 8.3 | 11.3 |
| 37 | 11 - 13 | 0.00 | 22.74 | 0.00 | 0.0002 | 0.00 | 9.0 | 20.1 |
| 13 | >16 | 0.62 | 0.37 | 0.00 | 0.0009 | 6.30 | 21.9 | 19.5 |
| 17 | >16 | 0.02 | 0.53 | 0.00 | 0.0014 | 6.82 | 11.1 | 7.1 |
| Average |  | 1.08 | 3.93 | 0.07 | 0.0521 | 4.37 | 12.3 | 13.7 |
| Drainage system |  |  |  |  |  |  |  |  |
| D1 | -- | 5.19 | 0.00 | 0.00 | 0.0319 | 0.00 | 9.7 | 5.4 |
| D2 | -- | 12.42 | 0.07 | 0.02 | 0.0159 | 0.24 | 12.9 | 6.1 |
| D3 | -- | 2.94 | 0.00 | 0.00 | 0.0043 | 0.00 | 11.4 | 7.0 |
| D4 | -- | 3.62 | 0.00 | 0.00 | 0.0010 | 0.00 | 12.7 | 7.6 |
| D5 | -- | 3.66 | 0.00 | 0.00 | 0.0028 | 0.01 | 12.4 | 7.9 |
| D6 | -- | 1.92 | 0.00 | 0.00 | 0.0013 | 0.04 | 10.8 | 5.9 |
| D7 | -- | 2.98 | 0.00 | 0.00 | 0.0020 | 0.18 | 12.2 | 7.6 |
| D8 | -- | 3.44 | 0.00 | 0.00 | 0.0021 | 0.00 | 12.4 | 7.8 |
| D9 | -- | 0.13 | 0.01 | 0.00 | 0.0006 | 0.16 | 5.7 | -1.3 |
| Average |  | 4.03 | 0.01 | 0.00 | 0.0069 | 0.07 | 11.1 | 6.0 |
